# Respiratory Metabolism and Antioxidant Response in Chinese Mitten Crab *Eriocheir sinensis* During Air Exposure and Subsequent Reimmersion

**DOI:** 10.3389/fphys.2019.00907

**Published:** 2019-07-17

**Authors:** Jie Bao, Xiaodong Li, Yuenan Xing, Chengcheng Feng, Hongbo Jiang

**Affiliations:** Liaoning Provincial Key Laboratory of Zoonosis, Department of Aquaculture, College of Animal Science and Veterinary Medicine, Shenyang Agricultural University, Shenyang, China

**Keywords:** *Eriocheir sinensis*, air exposure, reimmersion, oxygen consumption rate, ammonia excretion rate, O:N, respiratory metabolic enzyme, antioxidant enzyme

## Abstract

Chinese mitten crab, *Eriocheir sinensis*, often suffers from severe air exposure stress during transportation and culture; high mortality occurs due to desiccation. In this study, the effects of air exposure stress (0, 2, 4, 8, and 16 h) and reimmersion (2, 6, 12 h) on respiratory metabolism and antioxidant responses in Chinese mitten crabs were studied under laboratory conditions. The results showed that air exposure and reimmersion had a significant impact on the oxygen consumption rate (OCR), ammonia excretion rate (AER), oxygen to nitrogen ratio (O:N), superoxide dismutase (SOD), catalase (CAT), succinate dehydrogenase (SDH), and lactate dehydrogenase (LDH). Significant interaction between air exposure and reimmersion was observed for OCR, AER, O:N, SOD, CAT, SDH, and LDH in Chinese mitten crab. During the air exposure stage, SOD, CAT, and LDH activities in the gills and hepatopancreas first increased and then decreased as air exposure time increased. All of these parameters were significantly higher in the 4-h air exposure group than those in the control group. All the parameters were significantly lower in the 16-h air exposure group than those in the control group, except LDH in the hepatopancreas. However, SDH activity gradually decreased with increased air exposure time, and all the air exposure groups were markedly lower than those in the control group in the gills. During the reimmersion stage, OCR, AER, and O:N restored to normal levels after 12-h reimmersion, except in the 16-h air exposure group, where OCR and O:N were significantly higher than those in the control group and AER was significantly lower than that in the control group. The LDH activity in all groups restored to normal levels after 12-h reimmersion. The SDH, SOD, and CAT activities of the 2- and 4-h air-exposed groups returned to normal levels after 12-h reimmersion; however, these three parameters were still significantly higher in the 16-h air-exposed group than in the control group in the gills and hepatopancreas. Overall, Chinese mitten crabs reduce aerobic respiration and increase anaerobic respiration capacity during desiccation. Under air exposure stress, Chinese mitten crabs change their energy utilization mode to meet their energy demands and adjust their respiratory metabolism and antioxidant enzymes activities to adapt to adverse environments.

## Introduction

The Chinese mitten crab, *Eriocheir sinensis*, is one of the most important economic freshwater crustaceans in China. In 2016, the total aquaculture production of Chinese mitten crab was 800,000 tons, and its output value was over 50 billion Yuan (Bureau of Fisheries and Fishery Management of Chinese Ministry of Agriculture, 2016). In recent years, the natural resources of Chinese mitten crab have become severely depleted, while market demand has increased year by year. Therefore, demands on artificial culture have increased. To reduce transportation costs and avoid mutual attacks in water, live transport of larvae and adult Chinese mitten crab is done in dry conditions, without water. This transportation method exposes crabs to the air for relatively long periods. In the air-exposed state, the crabs are effectively unable to obtain oxygen, causing severe hypoxic stress. Hypoxia stress can hinder the metabolism of crustaceans and affect a series of physiological activities, as they adjust their physiological and biochemical indicators to adapt to environmental changes (Lehtonen and Burnett, 2016; Lucu and Ziegler, 2017). Studies have shown that long-term air exposure stress can disturb osmotic pressure in crustaceans and affect molting and growth (Sang and Fotedar, 2005; Mugnier et al., 2008). Even a short period of air exposure stress can affect the oxygen binding capacity of hemocyanin and hinder respiratory metabolism (Urbina et al., 2013).

Oxygen consumption rate (OCR), ammonia excretion rate (AER), and oxygen to nitrogen ratio (O:N) are important indicators of respiratory metabolism in aquatic animals. Respiratory metabolism can be used to evaluate the energy utilization patterns of aquatic animals under stress (Yang et al., 2006; Wang et al., 2011). Respiratory metabolism capacity is mainly reflected in the changes in respiratory metabolic enzyme activity. Aquatic animals usually respire aerobically, but they can provide energy for the body through anaerobic respiration under hypoxic stress (Paschke et al., 2010; Qiu et al., 2011). Mitochondria are the main sites of cellular respiratory metabolism, and their intima contains key enzymes in the respiratory chain. Succinate dehydrogenase (SDH), a mitochondrial marker enzyme, is located at the beginning of the respiratory chain and plays an important role in aerobic respiratory metabolism (Gao et al., 2016). As an important regulator of anaerobic glycolysis, lactate dehydrogenase (LDH) can catalyze pyruvate conversion to lactic acid (Carlsson and Gade, 1986). Therefore, the activities of SDH and LDH represent the degree of aerobic and anaerobic respiratory metabolism. Many molecules are produced during cell respiration, including peroxides, superoxide, hydroxyl radical, and singlet oxygen; they have strong activity and are known as reactive oxygen species (ROS) (McLennan and Degli, 2000). ROS play an important role in clearing away intrusive exogenous substances; however, excessive ROS has a destructive effect on organisms. Aquatic animals produce a large number of ROS in response to environmental stress (Walters et al., 2016). For this reason, organisms have evolved a set of antioxidant defense mechanisms to prevent oxidative stress damage caused by excessive ROS. Superoxide dismutase (SOD) and catalase (CAT) are considered to be important enzymes for removing ROS in crustaceans (Liu et al., 2018; Hong et al., 2019). Therefore, it is important to study the effects of air exposure stress on both respiratory metabolism and antioxidant responses in crustaceans to understand their full physiological responses to hypoxia stress. In this study, the changes in OCR, AER, and O:N of Chinese mitten crab during different air exposure stress and reimmersion times were examined. The activities of respiratory metabolic enzymes and antioxidant enzymes in gill and hepatopancreas were also measured. This study analyzed the air exposure adaptive mechanisms of Chinese mitten crab to provide information for transportation without water and aquaculture in crabs.

## Materials and Methods

### Ethics Statement

All animal experiments were conducted according to the China Government Principles for the Utilization and Care of Animals Used in Testing, Research, and Training (State science and technology commission of the People’s Republic of China). All protocols were approved by Animal Experiments Ethics Committee of Shenyang Agricultural University for the care and use of laboratory animals.

### Experimental Materials

Juvenile Chinese mitten crabs (about 1,300 individuals), obtained from Panjin Guanghe Crab Industry Co., Ltd., were transported to the laboratory at Shenyang Agricultural University and then cultured in 300 L circulation aquaria. During the period of temporary culture, the pH was 7.2 and the temperature was 18 ± 1°C. Continuous aeration was supplied to ensure sufficient dissolved oxygen, the DO was 7.8 ± 0.2 mg·L^−1^. The crabs were fed compound feed twice and we renewed 1/3 water daily. After 14 days temporary culture, healthy and energetic individuals with a body weight of 3.82 ± 0.16 g were randomly selected for the experiment.

### Experimental Design

The experiment was divided into two stages: air exposure stress and reimmersion after stress. Previous experiments showed that Chinese mitten crabs (similar size in present study) may die when exposed to air for 20 h, but that they can survive normally after exposure to air for 16 h (Bao et al., 2019). The experiment included five treatment groups of different air exposure times (0, 2, 4, 8, and 16 h, with 0 h as the control). There were four replicates of each group. During the air exposure stage, forty crabs were taken out of the circulation aquaria and dried with paper towel. Then, they were put in separate empty, dry aquaria (500 mm × 250 mm × 300 mm). At the end of each air exposure stress, eight crabs per dry aquarium were taken and anesthetized on ice for 5 min. Carapace was removed by using surgical scissor, and hepatopancreas and gills were collected for respiratory metabolic enzymes and antioxidant enzymes. Before reimmersion, two crabs were immediately taken from each aquarium and put into a bottle respirator to determine respiratory metabolism for 2 h (marked as reimmersion 1–2 h).

The remaining crabs in each treatment group were immediately returned to their circulation aquaria (500 mm × 250 mm × 300 mm) for reimmersion. The culture conditions were consistent with those in the temporary cultivation. During the reimmersion stage, crabs were measured at 2, 6, and 12 h after reimmersion. The sampling processing methods were the same as above. Respiratory metabolism was determined at 3–4 h, 7–8 h, and 13–14 h after reimmersion, separately.

### Determination of Parameters

#### Respiratory Metabolism

OCR and AER were measured using a hydrostatic respiratory system. The measurements were carried out in a 1-L triangular cone bottle. Two crabs were placed in each bottle. Four replicates for each time point of reimmersion were used, and four blank bottles (without crabs) were used as controls. The specific experimental operation can be found in Bao et al. (2018). The initial DO was 7.8 ± 0.1 mg·L^−1^, the water temperature was 18 ± 1°C, and respiratory metabolism was measured over 2 h. The initial and final DO and ammonia were determined using Winkler titration and the indophenol method, respectively. After the experiment, the surface water of each crab was absorbed using a paper towel, and the wet weight was determined immediately.

#### Respiratory Metabolic Enzyme Assay

After 0-, 2-, 4-, 8-, and 16-h air exposure stress and 2-, 6-, and 12-h reimmersion, crabs were randomly sampled and dissected. Samples of gills and hepatopancreas were taken from each individual and frozen in liquid nitrogen to analyze enzyme activities. Four replicates were measured for each time point. All samples were tested using analysis kits manufactured by Nanjing Jiancheng Bioengineering Institute (Nanjing, Jiangsu province, China). The activities of SDH (EC 1.3.5.1) and LDH (EC 1.1.1.27) were measured according to the manufacturer instructions. The measuring principles and sample processing method of SDH and LDH can be found in Bao et al. (2018).

#### Antioxidant Enzyme Analysis

The gill and hepatopancreas tissues were rinsed in 8.6 g·L^−1^ pre-cooled saline to remove the tissue fluid and then put in a centrifugal tube after being weighed. Normal saline was added for ultrasonic comminution according to *m* (tissue, g):*V* (normal saline, ml) = 1:9. The 10% tissue homogenate was centrifuged at 8,000*g* for 10 min at 4°C, and the supernatant was stored at −80°C to determine enzyme activity. Both SOD (EC 1.15.1.1) and CAT (EC 1.11.1.6) were determined using a kit from Nanjing Jiancheng Bioengineering Institute. The specific determination methods were carried out according to the manufacturer instructions. When the inhibition rate of SOD reached 50% in the reaction system, the corresponding amount of enzyme was defined as a unit of SOD activity (U·mg prot^−1^). A unit of CAT activity was defined as decomposition 1 μmol H_2_O_2_ per mg tissue protein per second (U·mg prot^−1^).

### Data Calculation and Statistical Analysis

The standard metabolic rate was evaluated in terms of OCR, AER, and O:N.

$$\mathrm{OCR}\;\left(\mathrm{mg}\cdot g^{-1}\cdot h^{-1}\right)\;\;=\;\;\frac{\left(\mathrm{DO}1-\mathrm{DO}2\right)\times V}{t\times W}$$

$$\mathrm{AER}\;\left(\mathrm{mg}\cdot g^{-1}\cdot h^{-1}\right)\;\;=\;\;\frac{\left(N2-N1\right)\times V}{t\times W}$$

$$O:N\;\;=\;\;\frac{14\times \mathrm{OCR}}{16\times \mathrm{AER}}$$

where, DO1 and DO2 are the initial and final oxygen concentrations of the metabolic experiment (mg O_2_·L^−1^), respectively, *V* is the volume of the test water (L), *t* is the duration of the test (h), *W* is the body weight of the crabs (g, wet weight), and N1 and N2 are the initial and final ammonia-nitrogen concentrations of the metabolic experiment (mg NH_3_-N·L^−1^), respectively. The O:N was expressed as the ratio of oxygen to nitrogen atoms.

The data were expressed as mean ± SE. A two-way ANOVA with SPSS 20.0 software was used to compare the differences for OCR, AER, O:N, SDH, LDH, SOD, and CAT activities between the air exposure and reimmersion treatment. Tukey’s *post hoc* test was used to make multiple comparisons, and *p* < 0.05 was taken as the significant difference level. All the data were tested for normality and homogeneity before ANOVA. When the homogeneity of variances was violated, a nonparametric test (the Welch test) was performed in this study.

## Results

### Effects of Air Exposure and Reimmersion on Respiratory Metabolism of Chinese Mitten Crab

**Figure 1:**
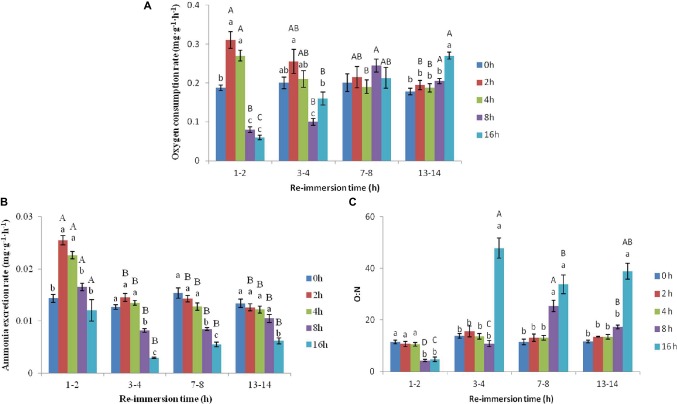
Effects of air exposure and reimmersion on oxygen consumption rate (OCR; **A**), ammonia excretion rate (AER; **B**), and oxygen to nitrogen ratio (O:N; **C**) in Chinese mitten crab. There was no significant difference between bars with the same letter (*p* < 0.05). Different lowercase letters represent a significant difference between the different air exposure times at the same reimmersion time (*p* < 0.05). Different uppercase letters represent a significant difference between the different reimmersion times at the same air exposure time (*p* < 0.05).

A two-way ANOVA showed that air exposure and reimmersion had a significant effect on the OCR (air exposure *F* = 15.733, *p* < 0.001; reimmersion *F* = 4.182, *p* = 0.009), AER (air exposure *F* = 101.907, *p* < 0.001; reimmersion *F* = 109.084, *p* < 0.001), and O:N (air exposure *F* = 90.441, *p* < 0.001; reimmersion *F* = 53.336, *p* < 0.001) of Chinese mitten crab. There was interaction effect on OCR, AER, and O:N between air exposure and reimmersion (OCR *F* = 15.027, *p* < 0.001; AER *F* = 8.949, *p* < 0.001; O:N *F* = 23.433, *p* < 0.001) (Figures 1A–C).

As shown in Figure 1A, the OCR after 2- and 4-h air exposure was significantly higher than that of the control group and was significantly lower after 8- and 16-h air exposure than that of the control group at 1–2 h reimmersion. As reimmersion time increased, the OCR began to decrease in the 2- and 4-h air exposure groups and returned to normal levels after 3–4 h reimmersion. In the 8-h air exposure group, OCR increased gradually and reached a maximum at 7–8 h reimmersion, then returned to the control level at the same time. The OCR continuously increased in the 16-h air exposure group and was significantly higher than that in the control group and other air exposure groups after 13–14 h reimmersion.

As shown in Figure 1B, the AER in the 2- and 4-h air exposure groups was significantly higher than that in the control group, while it was not different from the control group in the 8- and 16-h air exposure groups. As reimmersion time increased, the AER in the 2- and 4-h air exposure groups began to decrease and returned to normal levels after 3–4 h reimmersion. However, AER decreased and then increased in the 8- and 16-h air exposure groups and was the lowest after 3–4 h reimmersion, significantly lower than that in the control group. After 13–14 h reimmersion, the AER returned to the control group level in the 8-h air exposure group, but was still significantly lower than that in the control group in the 16-h air exposure group.

As shown in Figure 1C, the O:N in the 2- and 4-h air exposure groups was not significantly different from that in the control group at any time. However, the O:N in the 8- and 16-h air exposure groups was significantly lower than that in the control group after 1–2 h reimmersion. Subsequently, O:N in the 8- and 16-h air exposure groups began to increase and reached the highest value after 7–8 h and 3–4 h reimmersion. After 13–14 h reimmersion, the O:N after 16-h air exposure was still significantly higher than that in the control group, while that in the 8-h air exposure group returned to normal levels.

### Effects of Air Exposure and Reimmersion on Respiratory Metabolic Enzymes in Chinese Mitten Crab

#### Effects of Air Exposure and Reimmersion on SDH in Chinese Mitten Crab

**Figure 2:**
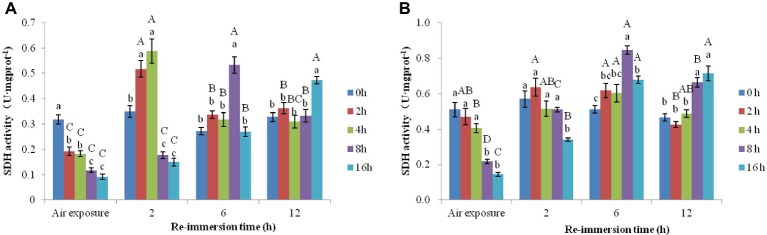
Effects of air exposure and reimmersion on succinate dehydrogenase (SDH) in the gills **(A)** and hepatopancreas **(B)** of Chinese mitten crab. Different lowercase letters represent a significant difference between the different air exposure times at the same reimmersion time (*p* < 0.05). Different uppercase letters represent a significant difference between the different reimmersion times at the same air exposure time (*p* < 0.05).

A two-way ANOVA revealed that air exposure and reimmersion significantly affected SDH activity in gills (air exposure *F* = 15.545, *p* = 0.003; reimmersion *F* = 75.208, *p* < 0.001) and hepatopancreas (air exposure *F* = 4.485, *p* < 0.001; reimmersion *F* = 75.618, *p* < 0.001) in Chinese mitten crab. There was significant interaction effect on gills and hepatopancreas between air exposure and reimmersion (gills *F* = 35.75, *p* < 0.001; hepatopancreas *F* = 20.979, *p* < 0.001) (Figures 2A,B).

As shown in Figure 2A, SDH activity in gills gradually decreased with increased air exposure time, and all the air exposure groups were significantly lower than the control group. After reimmersion, all air exposure groups showed an increase in SDH. In the 2- and 4-h air exposure groups, the highest SDH occurred after 2-h reimmersion, which was significantly higher than that of the control group. In the 8-h air exposure group, the activity of SDH was the highest after 6-h reimmersion and was significantly higher than that in the other groups. After 12-h reimmersion, the SDH activity in the 2-, 4-, and 8-h air exposure groups had returned to normal levels; however, the SDH activity in the 16-h air exposure group was still significantly higher than that in the control group.

As shown in Figure 2B, SDH activity in the hepatopancreas gradually decreased as air exposure time increased and was significantly lower than that of control group in the 8- and 16-h air exposure groups. After reimmersion, there was no significant difference in SDH activity between the 2- and 4-h air exposure and control group at any time. However, SDH activity in the 16-h air exposure group gradually increased as reimmersion time increased and was significantly higher than that in the control group after 6- and 12-h reimmersion.

#### Effects of Air Exposure and Reimmersion on LDH in Chinese Mitten Crab

**Figure 3:**
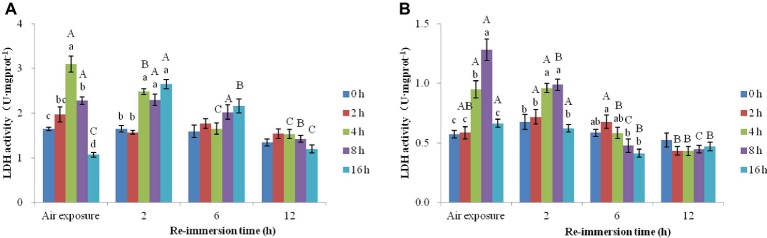
Effects of air exposure and reimmersion on lactate dehydrogenase (LDH) in the gills **(A)** and hepatopancreas **(B)** of Chinese mitten crab. Different lowercase letters represent a significant difference between the different air exposure times at the same reimmersion time (*p* < 0.05). Different uppercase letters represent a significant difference between the different reimmersion times at the same air exposure time (*p* < 0.05).

A two-way ANOVA showed that air exposure and reimmersion had a remarkable impact on LDH activities in gills (air exposure *F* = 20.314, *p* < 0.001; reimmersion *F* = 40.369, *p* < 0.001) and hepatopancreas (air exposure *F* = 18.473, *p* < 0.001; reimmersion *F* = 60.078, *p* < 0.001) in Chinese mitten crab. There was interaction effect on the gills and hepatopancreas between air exposure and reimmersion (gills *F* = 17.257, *p* < 0.001; hepatopancreas *F* = 11.708, *p* < 0.001) (Figures 3A,B).

Figure 3A shows that LDH activity in the gills was significantly higher than that in the control group in the 4- and 8-h air exposure groups and significantly lower than that in the control group in the 16-h air exposure group. After 2-h reimmersion, the activity of LDH in the 4-, 8-, and 16-h air exposure groups were significantly higher than that in the control group, while LDH in all the air exposure groups returned to normal levels after 6- and 12-h reimmersion.

Figure 3B shows that the LDH activity in the hepatopancreas was significantly lower in the control group than in the 4- and 8-h air exposure groups but was not significantly different from the 2- and 16-h air exposure groups. As in the gills, LDH in all the air exposure groups returned to normal levels after 12-h reimmersion.

### Effects of Air Exposure and Reimmersion on Antioxidant Enzymes in Chinese Mitten Crab

#### Effects of Air Exposure and Reimmersion on SOD in Chinese Mitten Crab

**Figure 4:**
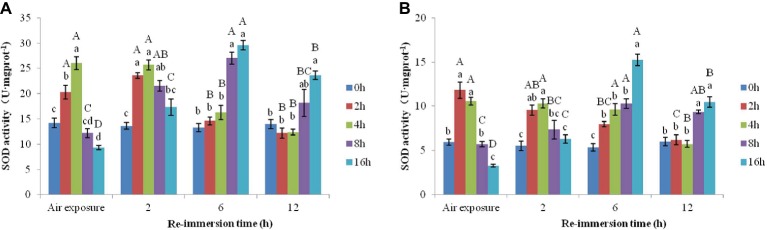
Effects of air exposure and reimmersion on superoxide dismutase (SOD) in the gills **(A)** and hepatopancreas **(B)** of Chinese mitten crab. Different lowercase letters represent a significant difference between the different air exposure times at the same reimmersion time (*p* < 0.05). Different uppercase letters represent a significant difference between the different reimmersion times at the same air exposure time (*p* < 0.05).

A two-way ANOVA revealed that SOD activities in gills (air exposure *F* = 22.193, *p* < 0.001; reimmersion *F* = 20.780, *p* < 0.001) and hepatopancreas (air exposure *F* = 26.790, *p* < 0.001; reimmersion *F* = 18.990, *p* < 0.001) in Chinese mitten crab were significantly affected by air exposure and reimmersion. And there was significant interaction for gills and hepatopancreas between air exposure and reimmersion (gills *F* = 31.043, *p* < 0.001; hepatopancreas *F* = 31.240, *p* < 0.001) (Figures 4A,B).

Figure 4A shows that the highest SOD activity in gills was observed in the 4-h air exposure group, which was significantly higher than that of the control group. The SOD activity in the 16-h air exposure group was significantly lower than that of the control group. After reimmersion, SOD activity in the 2- and 4-h air exposure groups were significantly higher than that in the control group after 2-h reimmersion and restored to normal levels after 6-h reimmersion. The 8- and 16-h air exposure groups showed an initial increase and then a decrease as reimmersion time increased. SOD reached its highest level after 6-h reimmersion, which was significantly higher than that in the control group. After 12-h reimmersion, only SOD in the 16-h air exposure group was significantly higher than that in the control group.

Figure 4B shows that the highest SOD activity in hepatopancreas was observed in the 2-h air exposure group, which was significantly higher than that in the control group. The SOD activity in the 16-h air exposure group was significantly lower than that in the control group. As reimmersion time increased, SOD showed a decreasing trend in the 2- and 4-h air exposure groups, which was significantly higher than that in the control group after 2- and 6-h reimmersion. SOD restored to normal levels after 12-h reimmersion. The 8- and 16-h air exposure groups showed an initial increase and then a decrease, with SOD reaching the highest levels after 6-h reimmersion, which were significantly higher than that in the control group. After 12-h reimmersion, the SOD activity in the 8- and 16-h air exposure groups were still significantly higher than that in the control group.

#### Effects of Air Exposure and Reimmersion on CAT of Chinese Mitten Crab

**Figure 5:**
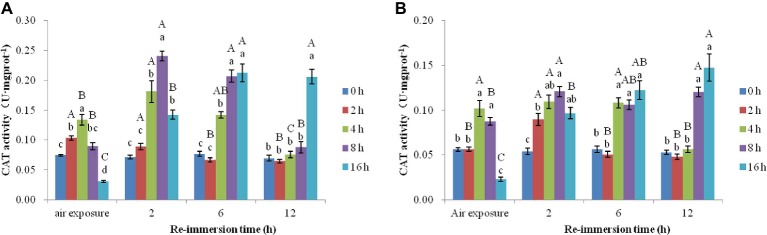
Effects of air exposure and reimmersion on catalase (CAT) in the gills **(A)** and hepatopancreas **(B)** of Chinese mitten crab. Different lowercase letters represent a significant difference between the different air exposure times at the same reimmersion time (*p* < 0.05). Different uppercase letters represent a significant difference between the different reimmersion times at the same air exposure time (*p* < 0.05).

A two-way ANOVA obtained that air exposure and reimmersion had a remarkable impact on CAT activities in gills (air exposure *F* = 90.129, *p* < 0.001; reimmersion *F* = 64.158, *p* < 0.001) and hepatopancreas (air exposure *F* = 58.351, *p* < 0.001; reimmersion *F* = 21.433, *p* < 0.001) in Chinese mitten crab. There was interaction effect on gills and hepatopancreas between air exposure and reimmersion (gills *F* = 42.891, *p* < 0.001; hepatopancreas *F* = 21.331, *p* < 0.001) (Figures 5A,B).

Figure 5A shows that the highest value of CAT activity in gills was found in the 4-h air exposure group. The CAT activity in the 2- and 4-h air exposure groups was significantly higher than that in the control group and was significantly lower in the 16-h air exposure group than that in the control group. As reimmersion time increased, CAT showed a decreasing trend in the 2-h air exposure group; however, the 4-, 8-, and 16-h air exposure groups showed an initial increase and then decrease. The highest level of CAT activity was observed in the 4- and 8-h air exposure groups after 2-h reimmersion and was significantly higher than that in the control group. The highest level of CAT in the 16-h air exposure group appeared after 6-h reimmersion. After 12-h immersion, CAT activity in all groups was restored to normal levels, except in the 16-h air exposure group, which remained significantly higher than in the control group.

Figure 5B shows that CAT activity in the hepatopancreas was significantly lower in the control group than that in the 4- and 8-h air exposure groups, but significantly higher than that in the 16-h air exposure group. After reimmersion, the highest values of CAT were observed in the 2-, 4-, and 8-h air exposure groups after 2-h reimmersion, while the highest value of CAT in the 16-h air exposure group was observed after 12-h reimmersion. After 12-h reimmersion, the CAT activity in the 8- and 16-h air exposure groups were still significantly higher than that of the control group, while CAT in the 2- and 4-h air exposure groups returned to normal levels.

## Discussion

When crustaceans encounter adverse environments, they adjust their physiological metabolic levels in response to the level of environmental stress (Leiva et al., 2018; Sun et al., 2018). Previous studies have shown that the OCR of crustaceans exposed to air is considerably less than that in water in the same conditions. However, oxygen consumption increases significantly on reimmersion in water (Defur et al., 1988; Taylor and Waldron, 1997). In this study, the OCR of Chinese mitten crabs significantly increased in the 2- and 4-h air exposure groups shortly after reimmersion, suggesting that oxygen debt accrued after air exposure stress. The rapid increase in OCR after reimmersion could have offset the large oxygen demand. However, the OCR in Chinese mitten crabs significantly decreased in the 8- and 16-h air exposure groups shortly after reimmersion. These results show that stress longer than 8 h in air exposure will induce metabolic depression. Similar result also found in *Cancer pagurus*, the hypometabolic level after air exposure stress was only 30% of when compared with the normoxic level for a resting crab (Regnault, 1992). As reimmersion time increased, OCR in the 2- and 4-h air exposure groups began to decrease and quickly returned to control levels. Conversely, the OCR in the 16-h air exposure group gradually increased and was significantly higher than that at preimmersion levels after 13–14 h reimmersion. Many crustaceans take around 8 h to recover to initial metabolic levels after air exposure (McMahon et al., 1979). From this study, we observed that the recovery time of oxygen consumption was not fixed. Whiteley and Taylor (1990) found that temperature was an important factor that affected the recovery period of *Homarus gammarus*. Our study confirmed that stress duration was also an important factor for crustaceans returning to standard metabolic levels. Studies have shown that the size of the oxygen debt is proportional to the duration of air exposure treatment (Bayne et al., 1976). These oxygen debts may come from reduced catabolic and anabolic metabolism, the oxygen resaturation of hemolymph, tissue water, mantle water, and possible “oxygen stores”(Widdows and Shick, 1985; Gnaiger and Staudigl, 1987). Therefore, large amounts of oxygen were involved in compensating oxygen debt after reimmersion, and it is hard to recover to the normal metabolic level in a short time for Chinese mitten crab.

Ammonia is the main form of nitrogen excretion in crustaceans, and the amount of urea and uric acid excretion is only 1–5% of total excretion (Weihrauch et al., 2009). Usually, after being exposed to air and hypoxia, the AER of crustaceans will decrease greatly. Conversely, blood and tissue ammonia will increase significantly and can be excreted rapidly after reimmersion, so AER will rise rapidly in the initial stage of reimmersion (Regnault, 1994). In this study, the AER of the 8- and 16-h air exposure groups did not change during 1–2 h reimmersion and significantly decreased after 3–4 h and 7–8 h reimmersion. This differs from *Cancer pagurus*, as Regnault (1994) found that AER was nearly 8-fold higher than the pre-emersion rate during the first 2 h of reimmersion. This supports the contention that ammonia had been stored in the body during air exposure stress. The results of our study showed a different situation as no such large ammonia efflux occurred rapidly upon reimmersion. Hong et al. (2007) found that Chinese mitten crabs can transform ammonia into urea and glutamine to avoid toxic effects on the body. We speculated that some other nitrogenous metabolic end products transformed from ammonia are formed and stored during long emersion times.

The O:N is widely used to estimate the metabolic energy supply of substrates (proteins, lipids, and carbohydrate) in aquatic animals (Tseng and Hwang, 2008). If energy is supplied by protein oxidation, the O:N is 3–16; if energy is supplied by equal protein and lipid oxidation, the O:N is 50–60; if energy is supplied by carbohydrate oxidation, the O:N is infinite (Mayzaud and Conover, 1988). Studies have shown that organisms can regulate energy utilization substrates when exposed to environmental stresses (Comoglio et al., 2005; Montagna and Collins, 2008). There are three main forms of utilization substrates that are regulated in crustaceans. The first form changes the energy supply from lipid and carbohydrate to protein. For example, when high-saline acclimated marine penaeid shrimp *Metapenaeus monoceros* were abruptly exposed to low-saline media, the dominant mode of metabolism shifted from lipid/carbohydrate toward protein (Pillai and Diwan, 2002). The second form changes the energy supply from protein to lipid and carbohydrate. For example, the freshwater crab *Trichodactylus borellianus* demonstrates this response when exposed to lower endosulfan solutions (Montagna and Collins, 2008). The third form occurs when higher proportions protein is recruited to maintain fundamental metabolism. For example, more protein is oxidized to maintain fundamental metabolism at higher metal concentrations in *Penaeus indicus* postlarvae (Chinni et al., 2000) and *Farfantepenaeus paulensis* (Barbieri, 2009). This indicates that the change in energy utilization mode is closely related to species, types, and duration of stress (Zhang et al., 2014; Chandurvelan et al., 2017). In this study, the O:N values of the 2- and 4-h air exposure groups were lower than 16 and were the same as that of the control group at all times, suggesting that protein was the main metabolic substrate in these groups. However, lipid became one of the important energy-using substances in the 8- and 16-h air exposure groups after 7–8 h and 13–14 h reimmersion. This is consistent with the second form outlined above. Bao et al. (2018) also found that Chinese mitten crabs used lipids as energy metabolic substrates after hypoxia treatment. This further shows that Chinese mitten crabs can adjust energy substances with changes in air exposure time and recruit more energy substances to meet energy demands after environmental stresses. The energy utilization mode after 12-h reimmersion did not return to the initial level, indicating that Chinese mitten crabs require a long period of physiological adjustment to cope with stress damage.

SDH and LDH are the marker enzymes of aerobic respiration and anaerobic respiratory metabolism, respectively (Simon and Robin, 1971; Viru, 1994). In this study, SDH activity in the gill tissue after air exposure was significantly lower than that in the control group. This indicated that air exposure stress induced cellular hypoxia in Chinese mitten crabs, which hindered electron transfer and ultimately decreased the efficiency of aerobic respiration metabolism. Conversely, LDH activity increased gradually as air exposure time increased and was significantly higher in the 4- and 8-h air exposure groups than that in the control group, indicating that the anaerobic respiratory metabolism of Chinese mitten crabs increased under air exposure stress. However, the LDH activity in gill tissues decreased significantly in the 16-h air exposure group. One reason might be that longer air exposure duration causes structural and functional damage to the gill tissue. In the initial stage of reimmersion, the time taken for SDH to return to normal levels in the 2- and 4-h air exposure groups was shorter than in the 8- and 16-h air exposure groups, which was consistent with the changes in OCR, AER, and O:N, indicating that respiratory metabolism requires more time to restore after a long period of air exposure.

Crustaceans have evolved a set of antioxidant enzymes, such as SOD and CAT, which remove excess ROS and protect the body from oxidative damage (Duan et al., 2015; Guo et al., 2017). Previous studies on *Litopenaeus vannamei* showed an increase in antioxidant activities during a period of hypoxia, which allowed them to tolerate environmental hypoxia and decrease oxidative stress (Li et al., 2016). This process was named “preparation for oxidative stress” (Hermes-Lima and Storey, 1998). Similar results were found in this study. SOD activity increased significantly after 2- and 4-h air exposure and remained significantly higher than that of the control group after 2-h reimmersion, indicating that Chinese mitten crab increased SOD activity to protect tissues against oxidative damage during air exposure and reimmersion. However, SOD decreased significantly after 16-h air exposure, which may indicate that excessive ROS produced from a long period of air exposure inhibited SOD activity (Wang et al., 2013). After reimmersion, the SOD activity increased gradually in the 16-h air exposure group and was significantly higher after 6- and 12-h reimmersion than that of the control group, indicating that there was still a large amount of ROS to be cleared after reimmersion. The accumulated ROS will likely have had adverse effects on these Chinese mitten crabs. In this study, the changes in trends of CAT were similar to those of SOD. After 12-h reimmersion, the CAT activity in the 16-h air exposure group was still significantly higher than that of the control group. The main reason might be that H_2_O_2_ will be produced primarily when SOD increases, which results in an increase in CAT to decompose H_2_O_2_ (Luna et al., 2004). Therefore, CAT activity still maintained at a high level after 12-h reimmersion. Compared with the hepatopancreas, the gill tissues had higher antioxidant enzyme activities, which was similar to results for the king crab *Paralomis granulosa* (Romero et al., 2011). We assume that the gill tissue plays an important role in the antioxidant defense of Chinese mitten crabs, but the specific mechanisms need to be further clarified.

## Conclusions

This study demonstrated that air exposure had important effects on respiratory metabolism and antioxidant responses in Chinese mitten crabs. After 2- and 4-h air exposure, the respiratory metabolism and antioxidant indices quickly returned to normal levels after reimmersion. However, after 16-h air exposure, the energy substrate shifted from protein to a protein-lipid mixture, SDH, SOD, and CAT did not return to normal levels, even after 12-h reimmersion. Overall, this indicates that the Chinese mitten crabs that experienced long periods of air exposure stress needed a long period of physiological adjustment to cope with stress damage and consumed more energy substances to meet energy demands. Therefore, the air exposure time should be shortened as much as possible in the process of transportation and culture of Chinese mitten crabs to reduce energy expenditure, and to avoid physiological stress and even death.

## Data Availability

The datasets generated for this study are available on request to the corresponding author.

## Ethics Statement

Experimental animal treatment methods were entirely consistent with the requirements of the Animal Experiments Ethics Committee of Shenyang Agricultural University.

## Author Contributions

JB and HJ were involved in designing of the research and wrote the manuscript. JB, YX, and CF performed the majority of the experiment, data processing, analysis, and interpretation. XL assisted in sample collection and detection of indicators. JB and HJ revised the manuscript.

### Conflict of Interest Statement

The authors declare that the research was conducted in the absence of any commercial or financial relationships that could be construed as a potential conflict of interest.
